# Identification of a Ferroptosis-Related Long Noncoding RNA Prognostic Signature and Its Predictive Ability to Immunotherapy in Hepatocellular Carcinoma

**DOI:** 10.3389/fgene.2021.682082

**Published:** 2021-10-21

**Authors:** Liang Wang, Xiangwei Ge, Zifeng Zhang, Yating Ye, Ziyi Zhou, Manhong Li, Hongxiang Yan, Lei Wu, Qian Bai, Jipeng Li, Jun Zhu, Yusheng Wang

**Affiliations:** ^1^ Department of Ophthalmology Eye Institute of Chinese PLA Xijing Hospital Fourth Military Medical University, Xi’an, China; ^2^ Department of Ophthalmology The Northern Theater Air Force Hospital, Shenyang, China; ^3^ Department of Oncology Chinese PLA General Hospital, Beijing, China; ^4^ College of Life Sciences Northwestern University, Xi’an, China; ^5^ The Hospital of 26th Base of PLA Strategic Support Force, Xi’an, China; ^6^ State Key Laboratory of Cancer Biology Institute of Digestive Diseases Xijing Hospital, Fourth Military Medical University, Xi’an, China; ^7^ Department of General Surgery The Southern Theater Air Force Hospital, Guangzhou, China

**Keywords:** ferroptosis, hepatocellular carcinoma, immune checkpoint blockers, lncRNA, prognosis

## Abstract

**Background:** Immune checkpoint blockers (ICBs) are increasingly being used to treat patients with advanced hepatocellular carcinoma (HCC), but only a third of these patients are sensitive to ICBs. Emerging evidence suggests that ferroptosis could be a novel target for antitumor treatment, and combined treatment with ferroptosis inducers might enhance sensitivity to immunotherapy. However, there is a lack of information on the crosstalk between ferroptosis-related lncRNAs and anti-tumor immunity. Therefore, we aim to explore prognostic value of ferroptosis-related lncRNAs and clarify potential role in ICBs of HCC.

**Methods:** We obtained mRNA and lncRNA expression data from two independent cohorts (TCGA and GEO database). Univariate Cox, the least absolute shrinkage and selection operator (Lasso) algorithm and multivariate Cox analysis were used to construct a lncRNA signature, which was evaluated using the area under the receiver operating characteristic curve (AUC) and Kaplan–Meier curves. Tumor-infiltrating cell (TIC) profiling and the tumor immune dysfunction and exclusion (TIDE) algorithm were used to validate the signature model and immunotherapy. Finally, we adopted RT-PCR assay to evaluate the differential expression of lncRNAs in HCC tissues in our hospital.

**Results:** The ferroptosis-related lncRNA signature included five lncRNAs, most of which were positively correlated with clinical stage and grade. The signature could stratify patients into two risk groups, with the high-risk group associated with a shorter overall survival (OS, *p* < 0.05) in TCGA-LIHC and GSE76427. Besides, the AUCs of the 1-, 3-, and 5-years OS were 0.772, 0.707, and 0.666, respectively. Gene set enrichment analysis (GESA) of lncRNAs revealed enrichment of oncogenic and immune-related pathways. The TIC profiling indicated a close correlation between the signature and immune cells. Furthermore, the high-risk group had a better response to immunotherapy than low-risk group. RT-PCR demonstrated these five lncRNAs were upregulated in cancerous tissue than normal tissues.

**Conclusions:** The ferroptosis-related lncRNA signature could accurately predict the OS of HCC patients and may serve as an independent clinical factor for patients’ outcomes. Ferroptosis-related lncRNAs may remodel the tumor microenvironment (TME) and affect the anti-cancer ability of ICBs, and therefore, could potentially act as an indicator for the response to immunotherapy in HCC.

## Introduction

Hepatocellular carcinoma (HCC) is one of the most common malignancies globally, contributing to one third of cancer mortalities (Bray et al., 2018). Although many recent advances have been made in the diagnosis and treatment of HCC, the overall survival (OS) rate for patients with HCC remains unsatisfactory. With the recent FDA approval of anti-programmed cell death-1 (PD-1) or anti-programmed cell death ligand-1 (PD-L1) antibodies (Keytruda, Tecentriq, nivolumab), immunotherapy and immune checkpoint blocker (ICB) therapies have gained increased attention as novel strategies for treating patients with HCC (Makarova-Rusher et al., 2015). ICBs have been used with increasing frequency for the treatment of patients with advanced HCC (Zhu et al., 2020a); however, only a subset of these patients can benefit from ICB treatment (Tang et al., 2020), possibly due to the complexity and heterogeneity of tumors, as well as many unknown variables in the tumor microenvironment (TME) (El Dika et al., 2019; Zhu et al., 2020b). The efficacy of immunotherapy could be impaired by complicated microenvironments, which contain various stromal cells and abundant immunosuppressive molecules (Prieto et al., 2015). Therefore, it is vital to explore the molecular mechanisms of HCC, and to develop a novel indicator for evaluating the response to ICBs, thus enabling optimization of treatment strategies.

Cell death is an essential part of most physiological and pathological processes (Vermeulen et al., 2003). Ferroptosis is a relatively recently discovered cell death process and is caused by an abnormal accumulation of iron-dependent lipid reactive oxygen species. Ferroptosis is distinct from other cell death processes such as necrosis, apoptosis, and autophagy (Dixon et al., 2012). Emerging evidence suggests that ferroptosis could be the target of innovative antitumor therapies (Xia et al., 2019; Liu et al., 2020; Du et al., 2021). Recent studies have found that immunotherapy induced CD8^+^ T cells could enhance ferroptosis by decreasing the expression of SLC7A11 complex (Wang et al., 2019a), which suggests that there is a relationship between ferroptosis in tumor cells and immune system activation. Meanwhile, the cancer cells experiencing ferroptosis might serve as arachidonic acid (AA) donors and affect anti-tumor immunity by participating in the production of bioactive immunomodulatory AA metabolites (Friedmann Angeli et al., 2019). Therefore, it is essential to study tumor immunotherapy from the perspective of the mechanism of ferroptosis.

Long noncoding RNAs (lncRNAs) are non-protein coding transcripts that are involved in a variety of complex biological processes (Quinn and Chang, 2016). Mounting evidence suggests that lncRNAs play essential roles in the occurrence and progression of different kinds of tumors, including HCC, by interacting with proteins or DNA and sponging miRNAs, as well as by encoding small bioactive peptides (Huang et al., 2020). Aberrant expression of lncRNAs is closely associated with tumor proliferation, apoptosis, migration, and invasion. LncRNA-ATB upregulates ZEB1 and ZEB2 by sponging miR-200, which may induce EMT and the invasion of HCC (Yuan et al., 2014). In addition, lncRNA CASC9 is highly expressed in HCC and may promote the proliferation of HCC by interacting with the protein HNRNPL (Klingenberg et al., 2018). However, little is known about the function of lncRNAs relating to ferroptosis in HCC. Therefore, the exploration of a ferroptosis-related lncRNA signature and its role in immunotherapy warrants urgent attention.

In the present study, a ferroptosis-related lncRNA signature in HCC was identified through correlation analysis, and a model was constructed based on five lncRNAs using multivariate Cox regression analysis. Next, we evaluated the model’s ability to predict the prognosis of patients with HCC independently and explored the reciprocal interaction between ferroptosis-related lncRNA and TME. Collectively, we found that ferroptosis-related lncRNAs could remodel TME and affect the anti-cancer ability of ICBs, and therefore, may be an ideal biomarker for evaluating the therapeutic effects of immunotherapy.

## Methods

### Demographic Characteristics

We downloaded transcriptome RNA sequencing (FPKM) data of 50 paracancerous and 374 cancerous HCC samples from The Cancer Genome Atlas (TCGA) Genomic Data Commons Data Portal (https://portal.gdc.cancer.gov/). Only 370 patients with complete and detailed following-up information were incorporated in our research. The “Limma” R software package was used to normalize the mRNA expression profile. The expression value of lncRNA was normalized using the quantile-normalized method. The corresponding clinicopathological features (age, sex, tumor differentiation grade, TNM stage, survival time, and survival state) were also obtained from TCGA database. The data from TCGA database is publicly available, and the present study was approved by the Ethics Committee of Xijing Hospital. The external cohort (GSE76427) with complete following-up information is from the Gene Expression Omnibus (GEO) database. Log2 transformation was conducted on this expression dataset (GSE76427).

### Ferroptosis-Related lncRNAs

Ferroptosis metagenes were retrieved from the FerrDb (http://www.zhounan.org/ferrdb/) repository, which is the first manually curated database for regulators and markers of ferroptosis and ferroptosis-disease associations (Zhou and Bao, 2020), including 259 ferroptosis genes. The relationship between lncRNAs and ferroptosis genes was calculated based on the RNA expression levels. Ferroptosis-related lncRNAs were identified using Spearman’s correlation coefficient, with an absolute value >0.4 and *p* < 0.0001 (Tang et al., 2021).

### Construction and Verification of a Ferroptosis-Related lncRNA Prognostic Signature (Fer-LPS)

To validate the predictive value of the ferroptosis-related lncRNA signature, we used univariate Cox proportional hazard regression analysis and determined 78 prognosis-related lncRNAs. Subsequently, we incorporated 78 prognosis-related lncRNAs into the least absolute shrinkage and selection operator (Lasso) method. The Lasso algorithm was performed for variable selection and shrinkage with the “glmnet” R package. Eventually, 11 ferroptosis-related lncRNAs validated by Lasso regression, were included in a multivariate Cox proportional hazard regression model based on the two‐step method. The final five lncRNAs and their responding coefficients (β) were verified, and the ferroptosis-related lncRNA prognostic signature (Fer-LPS) was constructed as follows: Risk scores = ∑(expression(lncRNAs)∗β) , where expression indicates lncRNA expression in HCC samples, and *β* represent its coefficients.

Using this equation, the HCC patients were divided into high-risk group (*n* = 185) and low-risk group (*n* = 185) based on the median cut-off value (0.9030). Kaplan–Meier (K-M) curves were plotted to explore the prognostic difference of high and low groups, which was tested by the log-rank test. The area under the curve (AUC) of receiver‐operating characteristic (ROC) curves were used to evaluate the predictive value of the Fer-LPS.

Univariate and multivariate Cox regression analyses of the available clinical variables were conducted to identify whether the risk score was an independent prognostic predictor of OS. Principal component analysis (PCA) was performed based on the expression of the Fer-LPS characteristic genes. The distribution of the groups was explored using t-distributed stochastic neighbor (t-SNE) analysis using the “Rtsne” R package.

### Functional Enrichment Analysis

Gene set enrichment analysis (GSEA) was performed to explore significant and underlying biological functions between the two risk groups (high‐ and low‐risk groups). Two well-known gene sets [h.all.v7.0. symbols.gmt (cancer hallmarks) and c7. all.v7.0. symbols.gmt (Immunologic signatures)] were downloaded from the Molecular Signatures database, and GSEA was performed (version 4.0.3). Gene set permutations were performed 1,000 times to achieve a normalized enrichment score (NES) for each analysis. A nominal *p* < 0.05 and false discovery rate (FDR) < 0.05 were considered to be statistically significant.

Gene Ontology (GO) and Kyoto Encyclopedia of Genes and Genomes (KEGG) enrichment analysis was used to explore the biological function of the differentially expressed genes (DEGs) between high- and low-risk groups. The DEGs were validated using the following cutoffs: |log2FC| ≥ 1 and FDR <0.05. The linear models for microarray data (LIMMA) R package was performed for DEGs identified between paracancerous and cancer samples as we reported before (Zhu et al., 2021). *p* values were adjusted using the Benjamini & Hochberg (BH) multiple testing correction.

### Profile of Tumor-Infiltrating Cells

Next, we estimated the tumor-infiltrating immune cell (TIC) abundance profiles and immune-related biological functions in a single sample GSEA (ssGSEA) algorithm (Rooney et al., 2015). Sixteen immune cell types were evaluated using TCGA RNA-seq data, including 7 T-cell subtypes, four dendritic cells (DCs), and five other immune cells [B cells, macrophages, natural killer cells (NK cells), neutrophils, and mast cells]. The immune-related pathways were calculated using ssGSEA in the “gsva” R package.

### Immunotherapeutic Response Prediction

PD-1, PD-L1 (Zongyi and Xiaowu, 2020), cytotoxic T-lymphocyte associated protein 4 (CTLA-4) (Agdashian et al., 2019), indoleamine 2,3-dioxygenase 1 (IDO1) (Li et al., 2017), lymphocyte-activation gene 3 (LAG3) (Anderson et al., 2016), T cell immunoreceptor with Ig and ITIM domains (TIGIT) (Anderson et al., 2016), and hepatitis A virus cellular receptor 2 (HAVCR2, also called TIM3) (Wolf et al., 2020) pathways are implicated in tumor immune evasion. Therefore, we analyzed the Spearman correlation between these immune checkpoints, their related molecules, and the Fer-LPS model. We used the tumor immune dysfunction and exclusion (TIDE, http://tide.dfci.harvard.edu/) algorithm (Jiang et al., 2018) and subclass mapping to predict clinical responses to ICBs (Lu et al., 2019; Xu et al., 2020). Patients whose TIDE prediction scores were more than zero were considered responders and other patients were considered non-responders.

### Statistical Analysis

All *p* values were two-tailed, and *p* < 0.05 was deemed to be statistically significant. K-M curve analysis with a log-rank test was used to compare the outcomes between different risk groups (low- and high-risk groups). Univariate and multivariate Cox regression analyses were adopted to identify independent clinical prognostic factors. A Mann-Whitney test was used to compare the ssGSEA scores of immune cells or functions between the two groups. All statistical analyses were conducted in R software (version 3.6.3). Related R packages including “ggplot2,” “ggpubr,” “stats,” “Rtsne,” “timeROC,” “glmnet,” “gsva,” “reshape2,” “survival,” and “survminer” were downloaded from the Lanzhou University Open-Source Society.

## Results

### Ferroptosis-Related lncRNAs in Hepatocellular Carcinoma

We extracted 259 ferroptosis-related genes from the FerrDb database (Zhou and Bao, 2020) (Supplementary Table S1). We identified lncRNAs that have a significant correlation with ferroptosis genes and obtained 242 ferroptosis-related lncRNAs, which were further used for the univariate Cox proportional hazard regression model.

### Construction of the Fer-LPS in Hepatocellular Carcinoma Survival

**FIGURE 1:**
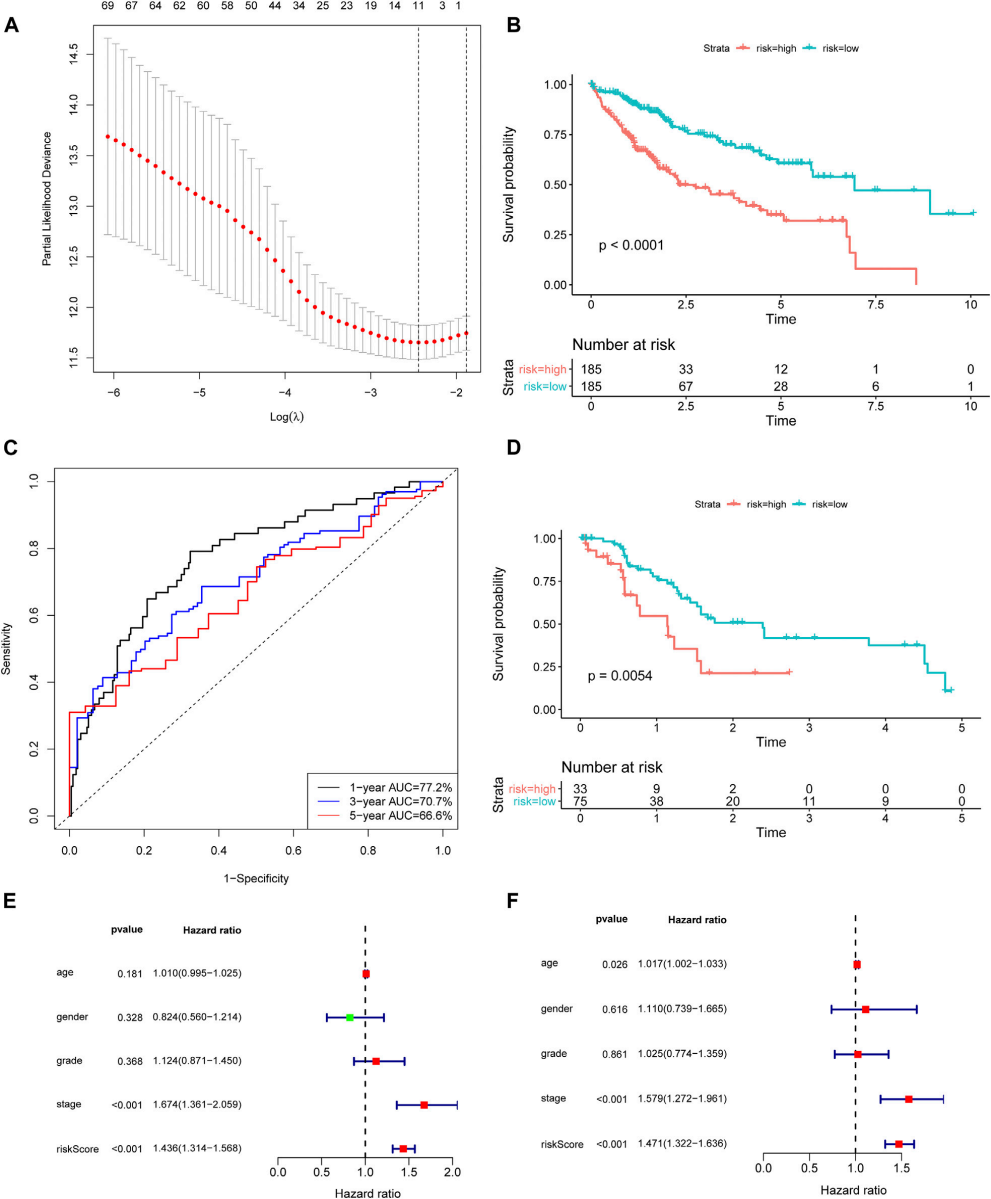
Construction and validation of the ferroptosis-related lncRNA signature. **(A)** Lasso coefficient profiles indicated that 11 ferroptosis-related lncRNAs should be retained in the model. **(B)** The K-M curve showed that the high-risk group (red curve) had a poorer OS than the low-risk group (blue curve) in TCGA cohort. **(C)** ROC curve of the ferroptosis-related lncRNA model. The AUCs of 1-, 3-, and 5 years OS were 0.772, 0.707, and 0.666, respectively. **(D)** The prognostic difference between high- and low-risk patients of GSE76427. **(E)** Univariate analysis and **(F)** Multivariate analysis of the lncRNA model and clinical features. lncRNA: long noncoding RNA; Lasso: least absolute shrinkage and selection operator; K-M curve: Kaplan–Meier curve; OS: overall survival; ROC curve: receiver‐operating characteristic curve.

Univariate Cox regression analysis demonstrated that 40 ferroptosis-related lncRNAs had a strong association with HCC outcomes (Supplementary Table S2). The Lasso algorithm identified an 11-lncRNA signature based on the optimal value of λ (Figure 1A). The final five ferroptosis‐related lncRNAs were identified using a multivariate Cox regression model for these ferroptosis‐related lncRNAs in the HCC cohort (Table 1). The risk score of Fer-LPS was calculated as follows: risk score = 0.3859 * LUCAT1 + 0.1757 * AC099850.3 + 0.2635 * AL365203.2 + 0.5739 * AL031985.3 + 0.2598 * AC009005.1.

**TABLE 1:** Multivariate COX regression analysis of ferroptosis-related lncRNA.

LncRNA	Coef	HR	95% CI	*p* Value
LUCAT1	0.3859	1.4710	1.1732–1.8444	0.0008
AC099850.3	0.1757	1.1921	0.9556–1.4871	0.1193
AL365203.2	0.2635	1.3015	1.0115–1.6745	0.0404
AL031985.3	0.5739	1.7752	1.0550–2.9871	0.0306
AC009005.1	0.2598	1.2967	1.0271–1.6370	0.0289

Coef: coefficients; HR: hazard ratio; *p* value was calculated by multivariate Cox analysis.

**FIGURE 2:**
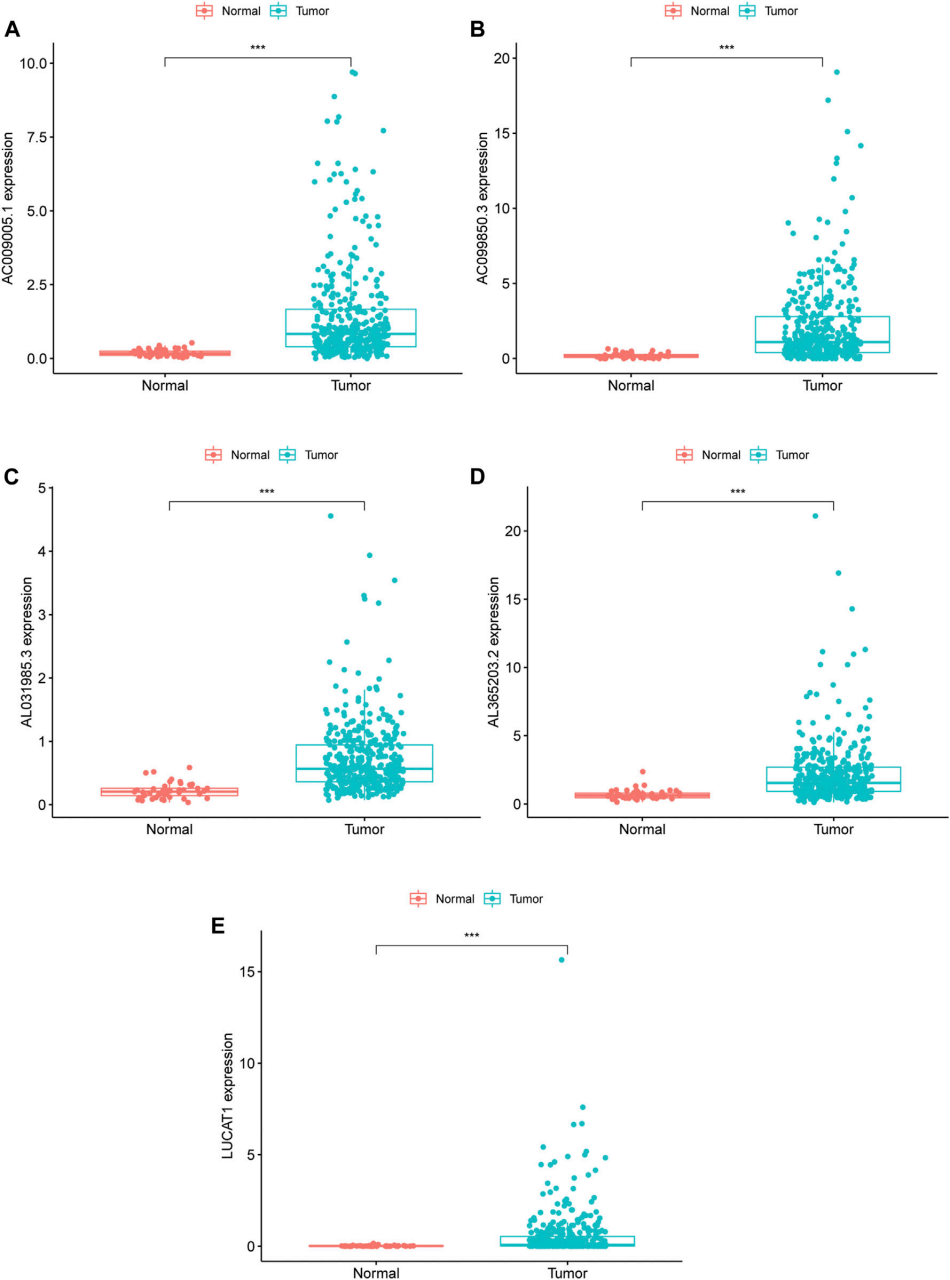
RNA expression of five ferroptosis-related lncRNA in TCGA database, **(A–E)** Differentially expressed lncRNA in tumor and adjacent normal tissues of HCC patients; **(A)** AC009005.1 **(B)** AC099850.3 **(C)** AL031985.3 **(D)** AL365203.2 **(E)** LUCAT1. ***means *p* value less than 0.001. HCC: hepatocellular carcinoma.

Based on the median value (0.9030) of the risk score, we divided HCC patients into high- and low-risk groups. The K-M curve indicated that high-risk group patients experienced a significantly worse OS than their low-risk counterparts (Figure 1B, *p* < 0.0001). The predictive performance of the risk score for OS was evaluated by time-dependent ROC curves, and the AUC reached 0.772 at 1 year, 0.707 at 3 years, and 0.666 at 5 years (Figure 1C). To further validate the robustness of Fer-LPS, we applied an independent cohort (GSE76427) from the GEO database. The K-M curve showed the low-risk patients possess better outcomes than their high-risk patients (Figure 1D, *p* < 0.0054). In addition, the expression levels of these five lncRNAs were significantly upregulated in cancerous tissue compared to in normal adjacent tissue (*p* < 0.001 for all; Figure 2). Patients with low expression of these five lncRNAs had a longer OS than patients with high expression (*p* ≤ 0.001 for all; Supplementary Figure S1). The association between ferroptosis genes and these five lncRNAs is summarized in Supplementary Table S3.

### Validation of the Prognostic Value of the Fer-LPS Signature

To assess whether the prognostic value of the Fer-LPS was independent of other available clinical variables, univariate and multivariate Cox regression analyses were performed using age, gender, grade, stage, and the Fer-LPS as covariables. Only stage [hazard ratio (HR) = 1.669, 95% confidence interval (CI): 1.357–2.053], T stage (HR = 1.649, 95%CI: 1.357–2.009) and risk score (HR = 1.432, 95%CI: 1.311–1.565) had a close relation with the clinical outcomes of HCC patients (Figure 1E). Moreover, M stage (HR = 1.383, 95%CI: 1.048–1.825) and the Fer-LPS (HR = 1.485, 95%CI: 1.333–1.656) were significantly associated with OS when adjusted for age, gender, grade, and stage (Figure 1F).

**FIGURE 3:**
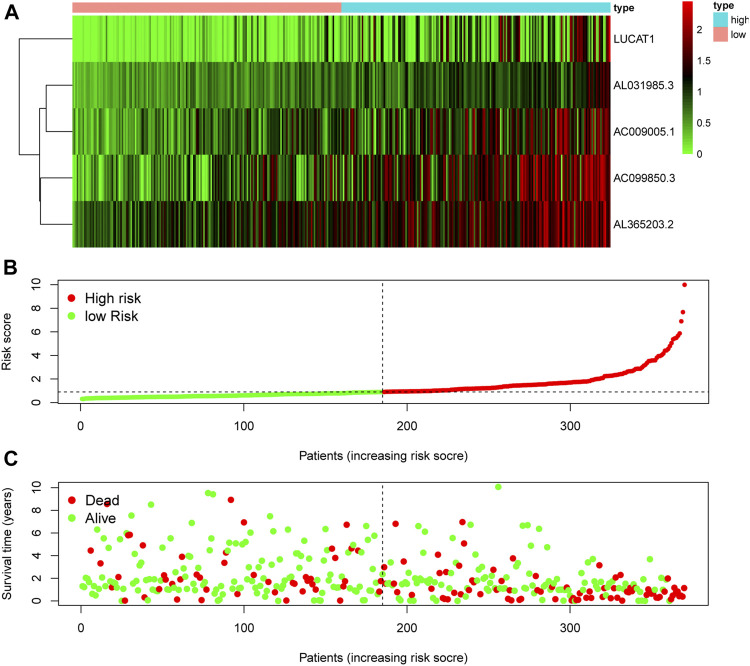
Evaluation of the ferroptosis-related lncRNA signature for prognostic prediction. **(A)** The expression pattern of five ferroptosis-related lncRNAs in low- and high-risk groups of HCC patients. **(B)** The risk score of each patient, showing grouping into low- and high-risk groups. **(C)** The relationship between OS and risk score for each patient. lncRNA: long noncoding RNA; OS: overall survival.

The RNA expression of five risky lncRNAs [lung cancer-associated transcript 1 (LUCAT1), AC099850.3, AL365203.2, AL031985.3, AC009005.1] was lower in the low-risk group than in the high-risk group (Figure 3A). The scatter diagram vividly showed the HCC patients in high-risk and low-risk groups (Figure 3B). As shown in Figure 2C, patients with a high risk had a higher probability of early death than those with a low risk.

**FIGURE 4:**
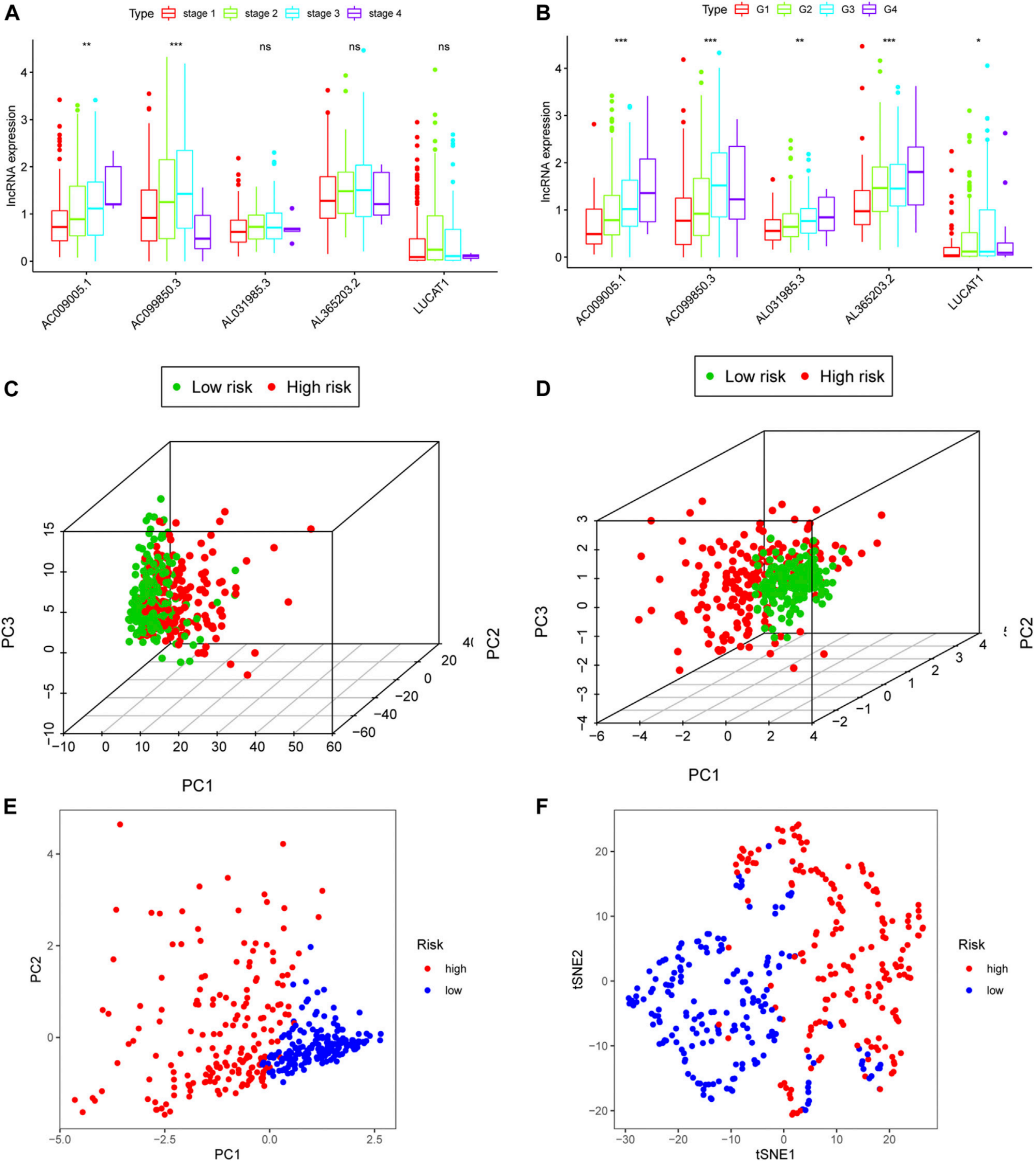
Clinical prognostic analysis of the five ferroptosis-related lncRNA models. **(A)** Relative expression of five ferroptosis-related lncRNAs in HCC patients at different clinical stages. **(B)** Relative expression of five lncRNAs in various cancer grades. **(C)** 3D-PCA plot of HCC patients divided into high- and low-risk groups based on gene expression (all genes). **(D)** 3D-PCA plot of HCC patients divided into high- and low-risk groups based on the expression of the five ferroptosis-related lncRNAs. **(E)** 2D-PCA plot of patients based on the expression of the five ferroptosis-related lncRNAs. **(F)** t-SNE plot of patients. lncRNA: long non-coding RNA; 3D/2D: three/two dimensional; PCA: principal component analysis.

To further explore the clinical role of each ferroptosis-related lncRNA in the proposed model, we compared the RNA expression of patients with different stages/grades of HCC. With advancing clinical stage, the expression of AC009005.1 and AC099850.3 increased significantly (Figure 4A). Similarly, all ferroptosis-related lncRNAs in the proposed model significantly changed with advancing grade (Figure 4B). Based on all the genes available in TCGA, the patients in different risk groups were distributed randomly in a PCA plot (Figure 4C). However, based on the five lncRNAs in the Fer-LPS model, PCA and t-SNE analysis indicated the patients in different risk groups were distributed in two clusters (Figures 4D–F).

### Functional Enrichment Analysis of the Fer-LPS

**FIGURE 5:**
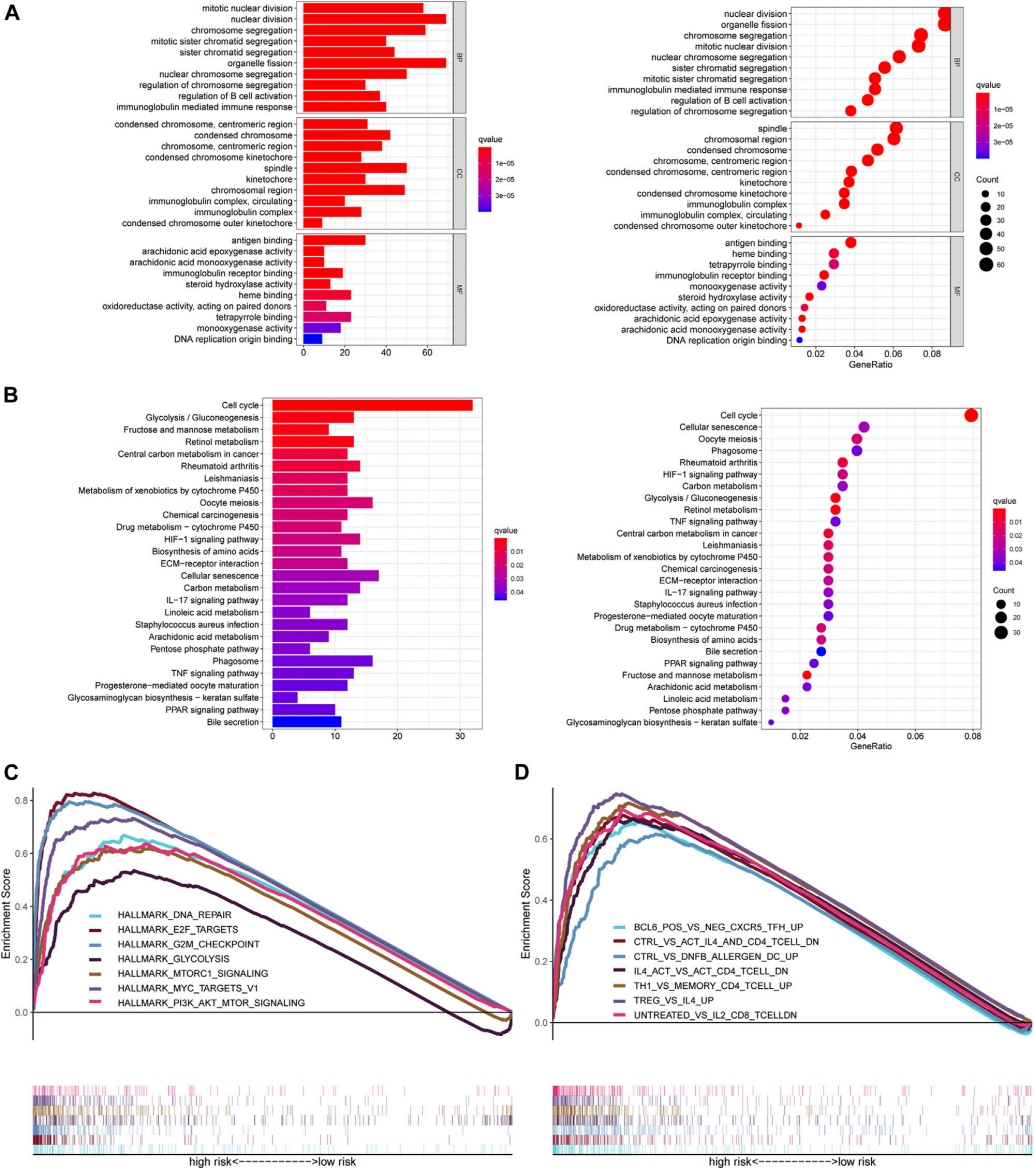
Functional analysis of the ferroptosis-related lncRNA signature. **(A)** GO enrichment analysis of ferroptosis-related lncRNAs. **(B)** KEGG pathway enrichment analysis of ferroptosis-related lncRNAs. **(C, D)** Several cancer hallmarks **(C)** and immunologic characteristics **(D)** are regulated via the immune‐related lncRNA signature. lncRNA: long noncoding RNA; GO: Gene Ontology; KEGG: Kyoto Encyclopedia of Genes and Genomes.

To further understand the molecular mechanisms of the ferroptosis‐related lncRNA signature, and how it is implicated in HCC progression, GO and KEGG analysis were performed. The top ten GO terms for biological processes were related to nuclear/chromosome segregation, B cell activation, and immunoglobulin mediated immune response. The top ten GO terms for the cellular components included condensed chromosome, centromeric region, spindle, and immunoglobulin complex. The top ten GO terms for molecular function were related to antigen binding, immunoglobulin receptor binding, and heme binding (Figure 5A). KEGG analysis confirmed that the ferroptosis‐related lncRNA signature was closely associated with the cell cycle processes, glycolysis, fructose and mannose metabolism, phagosome, HIF-1 signaling, IL-17 signaling, and TNF signaling (Figure 5B).

GSEA showed that the altered genes in the high-risk HCC patients belonged to pathways related to DNA repair signaling, E2F target signaling, G2M checkpoint signaling, glycolysis signaling, MTORC1 signaling, MYC target signaling, and PI3K AKT MTOR signaling (Figure 5C). Likewise, some immunity pathways were significantly enriched in the high-risk group, including pathways related to follicular helper T (Tfh) cells, CD4^+^ T cells, DC cells, CD8^+^ T cells, Th1 cells, and memory CD4 + T cells (Figure 5D). These results suggest that a high Fer-LPS risk score was linked with enhanced DNA repair, aberrant metabolic pathways, activation of classic tumor signal pathways, and immune evasion.

### Relationship Between the Fer-LPS and Tumor-Infiltrating Cells in Hepatocellular Carcinoma

**FIGURE 6:**
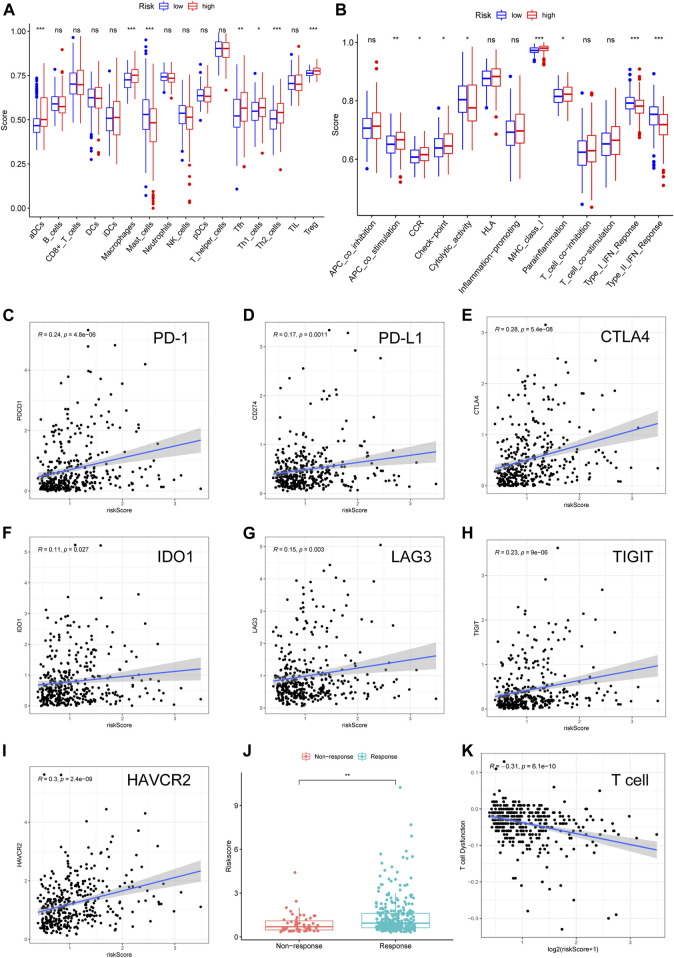
Validation of the ferroptosis-related lncRNA model in TICs and immunotherapy. The scores of **(A)** 16 immune cell subpopulations and **(B)** 13 immune-related functions varied in the high- and low-risk groups. **(C–I)** The correlation between seven important checkpoint molecules and the risk scores of HCC patients. **(J)** The relationship between risk score and response to immunotherapy was determined using the TIDE algorithm. **(K)** The relationship between T cell dysfunction and log2 (risk score). lncRNA: long noncoding RNA; TIC: tumor-infiltrating immune cells; TIDE: tumor immune dysfunction and exclusion. Log2: data log2 transformed.

Considering that several immune-related signaling pathways were enriched in the high-risk HCC group, we quantified the enrichment scores of diverse immune cell subpopulations, related functions, or pathways using ssGSEA to further explore the correlation between the risk score and the immune system. Interestingly, some TICs [dendritic cells (DCs), macrophages, mast cells, Tfh cells, Th1 cells, Th2 cells, and regulatory T (Treg) cells] were significantly different between the low- and high-risk groups in TCGA cohort (adjusted *p* < 0.05, Figure 6A). Among the TICs with differential profiles, some immunosuppression-related cells (macrophages and Treg cells) were more abundant in the high-risk group, while mast cells were less abundant in the high-risk group, compared to in the low-risk group. The cytokine-cytokine receptor (CCR), checkpoint, MHC class I, and parainflammation scores were higher in the high-risk group than in the low-risk group, while the activity of MHC cytolytic activity and type I/II IFN response were lower in the high-risk group than in the low-risk group (adjusted *p* < 0.05, Figure 6B). These results suggest that the Fer-LPS is associated with TICs in HCC.

### Correlation Between the Ferroptosis-Related lncRNA Signature and ICB Therapy Outcomes

A correlation between ferroptosis-related lncRNAs, TICs, and immunologic characteristics was identified. Based on these findings, we further investigated the role of these lncRNAs in ICBs therapy. Intriguingly, we found that the ferroptosis-related lncRNA signature was positively related to PD-1 (R = 0.24), PD-L1 (R = 0.17), CTLA-4 (R = 0.26), IDO1 (R = 0.11), LAG3 (R = 0.15), TIGIT (R = 0.23), and HAVCR2 (R = 0.3) (Figures 6C–I; all *p* values < 0.05). This suggests that more immune escape and more protein expression of immune checkpoints can be observed in the patients with high-risk scores. Furthermore, we found that the patients who did not respond to ICBs had a lower risk score than patients who responded to ICBs (Figure 6J), which suggests that high-risk patients would benefit from immunotherapy. Finally, we found that the risk score was significantly correlated with T cell dysfunction (R = −0.31, Figure 6K), which indicated that the TME of these two groups varied significantly.

### Validation of the Fer-LPS in Hepatocellular Carcinoma Tissues

**FIGURE 7:**
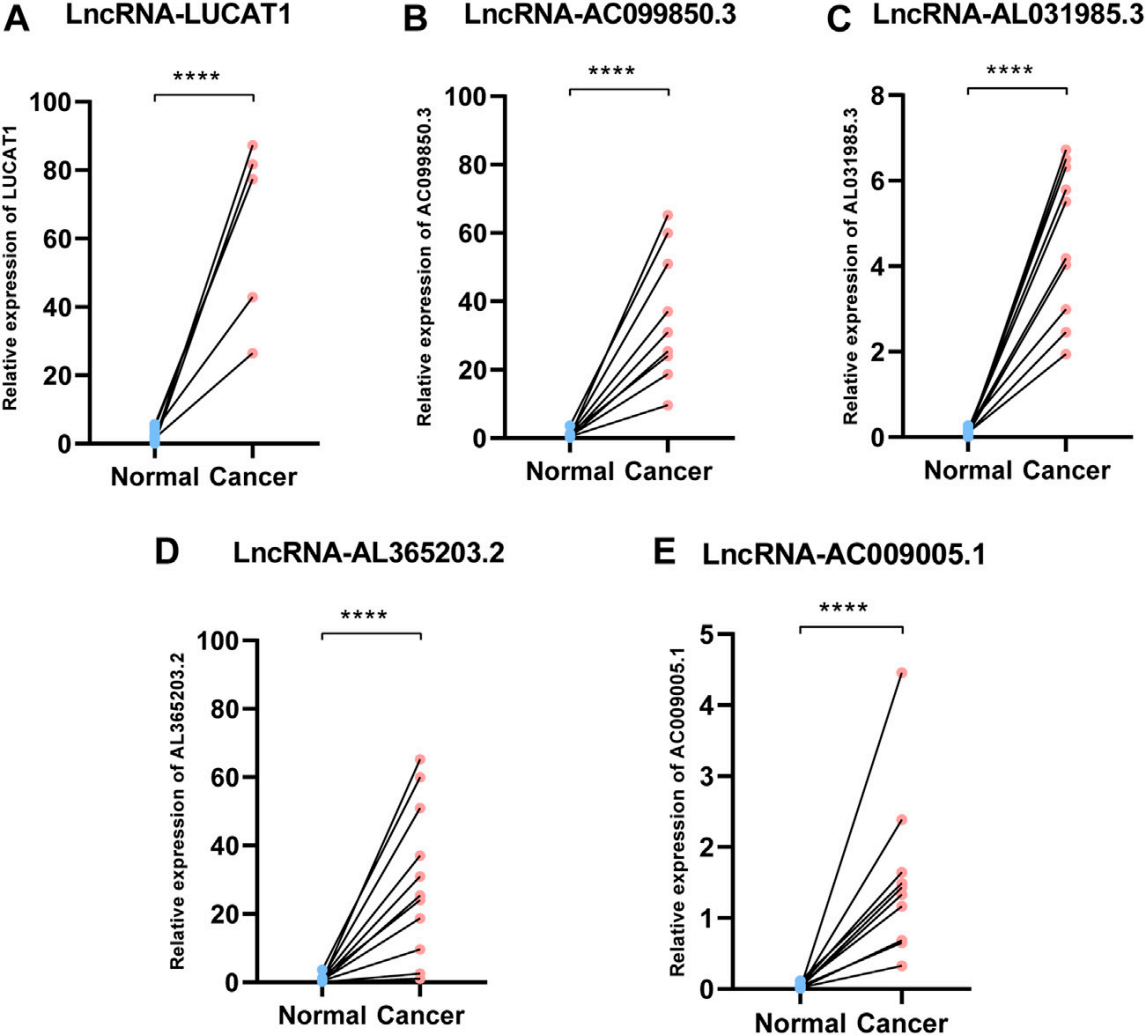
Expression of ferroptosis-related lncRNAs in HCC patients. Relative of RNA expression of five ferroptosis-related lncRNAs between cancerous and adjacent normal tissues. **(A)** LUCAT1 **(B)** AC099850.3 **(C)** AL031985.3 **(D)** AL365203.2 **(E)** AC009005.1. ****means *p* < 0.0001; HCC: hepatocellular carcinoma.

We collected ten pairs of tumor tissues and paratumor tissues in Xijing Hospital. After extracting RNA of tissues and RT-PCR, we found that these five ferroptosis-related lncRNAs (LUCAT1, AC099850.3, AL365203.2, AL031985.3, AC009005.1) were upregulated in cancerous tissues than normal adjacent tissues (all *p* < 0.001; Figure 7), which consistent to the results from TCGA database (Figure 2).

## Discussion

Although numerous therapies, including surgery, radiotherapy, and targeted therapies (tyrosine‐kinase inhibitors), have been comprehensively adopted for the treatment of HCC, the 5-years overall survival and life-quality of the patients with HCC has not been significantly improved. Phase II data highlighted the potential durable objective responses with ICB, prompting conditional FDA approval of nivolumab and pembrolizumab as second-line agents for treating HCC (Bangaru et al., 2020). Emerging evidence suggests that ferroptosis plays an essential role in immunotherapy and radiotherapy in HCC (Lang et al., 2019). Furthermore, a novel ferroptosis-related gene signature was identified for predicting survival rate in HCC patients and was positively associated with the TME (Liang et al., 2020). Although a few ferroptosis genes have proved clinically valuable in HCC patients, the potential mechanisms remain poorly understood and warranted further investigation. In the present study, we systematically investigated the expression of 242 ferroptosis-related lncRNAs in HCC tumor tissue and determined their predictive ability for OS. First, we constructed a novel predictive model integrating five ferroptosis-related lncRNAs and then validated it using ROC curves. External cohort (GSE76427) also validated the robustness of the Fer-LPS. Functional analysis revealed that immune-related pathways were enriched in a group of HCC patients that could be designated as “high-risk” based on our predictive model. Moreover, by TIDE algorithm, we found that the lncRNA model could be an indicator for the response to immunotherapy.

LncRNAs have been associated with the occurrence and progression of various types of cancer, including ferroptosis-related lncRNAs. The cytosolic lncRNA P53RRA, as a G3BP1-interacting lncRNA, could promote ferroptosis and apoptosis in cancer cells by regulating nuclear sequestration of p53 (Mao et al., 2018). In a study on erastin (a ferroptosis inducer), lncRNA GABPB1-AS1 was upregulated, which may consequently downregulate GABPB1 protein levels, thereby leading to a reduction in peroxiredoxin-5 (PRDX5) peroxidase levels and the eventual suppression of the cellular antioxidant response (Qi et al., 2019). A recent report showed that LINC00336 could serve as an endogenous sponge of microRNA 6,852 to regulate the expression of cystathionine-β-synthase (CBS), which in turn promotes ferroptosis (Wang et al., 2019b). High expression of nuclear factor erythroid 2-related factor 2 (NRF2) is an antioxidant transcript factor that protects malignant cells from ferroptosis. A novel strategy was identified to enhance erastin-induced ferroptosis in NSCLCs acting through the MT1DP/miR-365a-3p/NRF2 axis (Gai et al., 2020). Therefore, cancer-related lncRNAs have a vital role in tumorigenesis and ferroptosis.

In the present study, we first identified 242 ferroptosis-related lncRNAs. We then constructed a five lncRNA signature that could classify HCC patients into high- and low-risk groups with significantly different OS. We evaluated the predictive ability of our model and demonstrated that the Fer-LPS model could be an independent prediction indicator in HCC patients after correction for traditional clinical risk factors (stage or grade). Among the five ferroptosis-related lncRNAs in the model, LUCAT1 has been confirmed to be effective in promoting the progressions of various cancers. In colorectal cancer, patients with higher LUCAT1 showed worse prognosis and were less sensitive to chemotherapy (Huan et al., 2020). Another report indicated that LUCAT1 promotes the proliferation and metastasis of HCC cells *in vitro* and *in vivo* (Lou et al., 2019). However, there is no research on the relationship between LUCAT1 and ferroptosis in HCC, which warrants further investigations. AC099850.3 and AL365203.2 are autophagy-related lncRNAs included in our model and could be prognostic factors in HCC patients (Jia et al., 2020). AL031985.3 was previously identified as an immune-related prognostic lncRNA in HCC patients (Kong et al., 2020), which is consistent with our findings.

A large number of immune-related biological processes and pathways were enriched in the high-risk group, possibly because antigens released by ferroptotic cells were different from those released by other cancer cells (Friedmann Angeli et al., 2019). We further dissected the landscape of immune cells and related immune pathways in each risk group and found that Tregs and macrophages were more abundant in the high-risk group. Accumulating evidence suggests that tumor-associated macrophages (TAMs) may express cytokines and chemokines that can suppress anti-tumor immunity and promote tumor progression (Pathria et al., 2019); therefore, TAMs are considered as novel therapeutic targets (Komohara et al., 2016). Likewise, as definitive immunosuppressive cells, the entry of Treg cells into tumor tissue is often associated with poor prognosis. The combination of therapy targeting Treg cells (e.g., by reducing the number of Treg cells or attenuating their suppressive activity in tumor tissue) and the activation of tumor-specific effector T cells can potentially enhance cancer immunotherapy (Tanaka and Sakaguchi, 2017). The proportion of mast cells was lower in the high-risk group, which is consistent with previous reports. A recent study elucidated that in HCC patients, the proportion of activated mast cells was lower in cancer tissue than in the adjacent tissue or even in the healthy population (Rohr-Udilova et al., 2018). According to our model, higher risk scores were associated with impaired anti-tumor immunity, including the type I and II IFN responses. Similarly, LUCAT1 acts as a post-induction feedback regulator, which might restrain the immune response (Agarwal et al., 2020). Therefore, enhanced immunity escape in patients with high risk may explain the lower precision when predicting their prognosis. Finally, there were significantly more high-risk patients with positive response to treatment than non-responders, which means high-risk patients may experience stronger immune inhibition and may be more sensitive to immunotherapy, especially ICBs.

There were also some limitations to the present study. First, the research was based on a publicly available database, and more prospective real-world data is needed to verify the clinical utility of our model. Second, we could not clarify the regulatory mechanisms underlying the effects of these lncRNAs, and additional experimental research is required to explore this.

In conclusion, we have provided novel insights into ferroptosis-related lncRNA functions and constructed a new prognosis-related lncRNA signature with robust predictive value in HCC outcomes. We have elucidated the roles of ferroptosis-related lncRNAs in immunotherapy of HCC. These five lncRNAs may improve outcome predictions and guide clinical decision making for patients with HCC.

## Data Availability

The original contributions presented in the study are included in the article/Supplementary Material, further inquiries can be directed to the corresponding authors.
