# Supplementary figures and images for: Identification of a Ferroptosis-Related Long Noncoding RNA Prognostic Signature and Its Predictive Ability to Immunotherapy in Hepatocellular Carcinoma
Source: Front Genet. 2021 Oct 21;12:682082. doi: 10.3389/fgene.2021.682082 (PMC8566703; doi:10.3389/fgene.2021.682082)

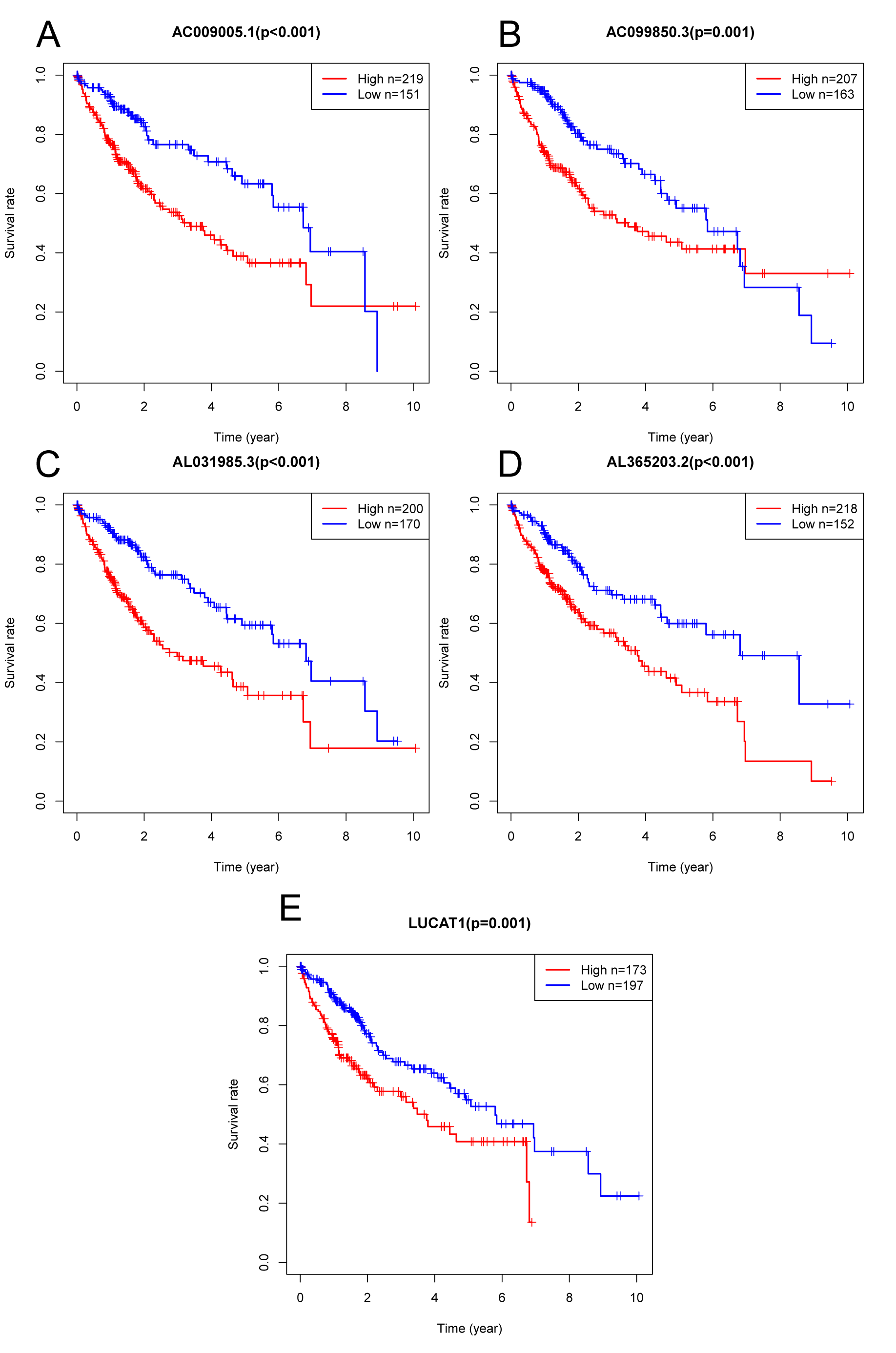

Supplement: Supplementary file 2 [file Image1.TIF]
