# Supplementary material for: Identification of a Ferroptosis-Related Long Noncoding RNA Prognostic Signature and Its Predictive Ability to Immunotherapy in Hepatocellular Carcinoma
Source: Front Genet. 2021 Oct 21;12:682082. doi: 10.3389/fgene.2021.682082 (PMC8566703; doi:10.3389/fgene.2021.682082)
Supplement: Supplementary file 3 [file Table2.docx]

Table S2: Univariate COX regression analysis of ferroptosis-related lncRNA

| id | HR | HR.95L | HR.95H | P value |
| --- | --- | --- | --- | --- |
| AC016405.3 | 1.308463 | 1.083932 | 1.579504 | 0.005125 |
| MAFG-DT | 1.387296 | 1.160523 | 1.658383 | 0.000325 |
| CYTOR | 1.330564 | 1.127007 | 1.570888 | 0.000748 |
| AC074117.1 | 1.888706 | 1.285921 | 2.77405 | 0.001186 |
| AL512598.1 | 1.396125 | 1.084262 | 1.797687 | 0.009676 |
| PCAT6 | 1.394077 | 1.124619 | 1.728096 | 0.002432 |
| AC073611.1 | 1.909517 | 1.322539 | 2.757011 | 0.000557 |
| BACE1-AS | 1.772923 | 1.286102 | 2.444017 | 0.000472 |
| ZEB1-AS1 | 2.144242 | 1.427299 | 3.221311 | 0.000239 |
| AL606489.1 | 1.457923 | 1.098527 | 1.934899 | 0.009036 |
| PIK3CD-AS2 | 1.539563 | 1.227564 | 1.93086 | 0.000188 |
| MAPKAPK5-AS1 | 1.482058 | 1.131996 | 1.940374 | 0.004212 |
| AC145343.1 | 1.633183 | 1.189071 | 2.243167 | 0.00245 |
| AC006504.8 | 1.783786 | 1.263241 | 2.518834 | 0.001012 |
| AC068473.5 | 1.763078 | 1.225501 | 2.53647 | 0.002245 |
| LUCAT1 | 1.691657 | 1.357737 | 2.107701 | 2.79E-06 |
| SNHG1 | 1.348184 | 1.076702 | 1.688117 | 0.00921 |
| THUMPD3-AS1 | 1.849547 | 1.288707 | 2.654463 | 0.00085 |
| AC009403.1 | 1.730131 | 1.253225 | 2.388518 | 0.000863 |
| LINC00665 | 1.305786 | 1.071755 | 1.590921 | 0.008106 |
| DANCR | 1.297285 | 1.110905 | 1.514934 | 0.001005 |
| AC020915.3 | 1.757151 | 1.212323 | 2.546828 | 0.002914 |
| AL139384.1 | 1.639214 | 1.216872 | 2.20814 | 0.001149 |
| AC093227.1 | 1.863093 | 1.224185 | 2.835451 | 0.003684 |
| AC005332.5 | 1.535801 | 1.147573 | 2.055367 | 0.003904 |
| AC012146.1 | 1.357964 | 1.087615 | 1.695515 | 0.006904 |
| AC007405.3 | 1.530604 | 1.173339 | 1.996652 | 0.001697 |
| LINC01273 | 1.578236 | 1.150491 | 2.165014 | 0.004667 |
| AC010761.1 | 1.688295 | 1.148147 | 2.482557 | 0.007763 |
| AC084033.3 | 1.419823 | 1.118304 | 1.802638 | 0.004002 |
| AC099850.3 | 1.523054 | 1.273426 | 1.821615 | 4.10E-06 |
| SNHG12 | 1.459707 | 1.101877 | 1.93374 | 0.008386 |
| SNHG21 | 1.991625 | 1.211585 | 3.273867 | 0.006591 |
| AC009779.2 | 1.820679 | 1.318654 | 2.51383 | 0.000272 |
| AL603839.3 | 1.606098 | 1.162689 | 2.218607 | 0.004048 |
| AC026401.3 | 1.464158 | 1.18362 | 1.81119 | 0.000443 |
| AL365203.2 | 1.654068 | 1.313062 | 2.083635 | 1.94E-05 |
| AC083799.1 | 1.514503 | 1.118008 | 2.051611 | 0.007357 |
| TMEM220-AS1 | 0.548242 | 0.414118 | 0.725806 | 2.68E-05 |
| AC004656.1 | 1.59106 | 1.216603 | 2.080769 | 0.000694 |
| AC016747.1 | 1.696879 | 1.201382 | 2.396739 | 0.002688 |
| AL031985.3 | 3.204513 | 2.176548 | 4.717979 | 3.62E-09 |
| LINC02362 | 0.741766 | 0.614422 | 0.895502 | 0.001881 |
| AL049840.4 | 1.453774 | 1.128362 | 1.873032 | 0.003803 |
| LINC00221 | 1.244213 | 1.058597 | 1.462375 | 0.00803 |
| NCK1-DT | 1.976303 | 1.2436 | 3.140697 | 0.003946 |
| MIR210HG | 1.518554 | 1.251104 | 1.843176 | 2.37E-05 |
| PRRT3-AS1 | 1.425151 | 1.183789 | 1.715723 | 0.000182 |
| AC124798.1 | 1.573719 | 1.253134 | 1.976318 | 9.56E-05 |
| SREBF2-AS1 | 1.945316 | 1.326878 | 2.851997 | 0.000652 |
| AC004816.1 | 1.626611 | 1.239556 | 2.134526 | 0.00045 |
| AC092171.2 | 1.373131 | 1.103396 | 1.708806 | 0.004487 |
| LINC01138 | 1.996328 | 1.419908 | 2.80675 | 6.99E-05 |
| NRAV | 2.049958 | 1.539017 | 2.730526 | 9.22E-07 |
| MIR4435-2HG | 1.447438 | 1.137639 | 1.8416 | 0.002618 |
| ELFN1-AS1 | 1.249036 | 1.078154 | 1.447003 | 0.003052 |
| AP003469.4 | 1.430783 | 1.119179 | 1.829144 | 0.004258 |
| F11-AS1 | 0.67476 | 0.530204 | 0.858729 | 0.001383 |
| ZFPM2-AS1 | 1.440275 | 1.239037 | 1.674196 | 2.02E-06 |
| AC115619.1 | 0.808857 | 0.716716 | 0.912844 | 0.000587 |
| SNHG20 | 1.646502 | 1.135852 | 2.386728 | 0.008478 |
| LINC00942 | 1.27657 | 1.122623 | 1.451628 | 0.000196 |
| AL050341.2 | 1.489583 | 1.119182 | 1.982572 | 0.006297 |
| SNHG3 | 1.467448 | 1.216881 | 1.769609 | 5.95E-05 |
| AC012467.2 | 2.005542 | 1.270301 | 3.166335 | 0.002819 |
| FOXD2-AS1 | 1.490096 | 1.18402 | 1.875295 | 0.000674 |
| WAC-AS1 | 1.739666 | 1.295537 | 2.336048 | 0.000232 |
| MSC-AS1 | 1.657905 | 1.332646 | 2.06255 | 5.70E-06 |
| AC009005.1 | 1.56699 | 1.26076 | 1.9476 | 5.15E-05 |
| LYRM4-AS1 | 1.885334 | 1.286905 | 2.762041 | 0.001135 |
| AC245297.3 | 1.45104 | 1.122762 | 1.875301 | 0.004444 |
| C8orf49 | 1.70373 | 1.150796 | 2.522337 | 0.007778 |
| AC145207.5 | 2.348174 | 1.549867 | 3.557674 | 5.65E-05 |
| AL139260.1 | 1.473182 | 1.13415 | 1.91356 | 0.003692 |
| CASC19 | 1.393305 | 1.137876 | 1.706072 | 0.001327 |
| SBF2-AS1 | 1.92984 | 1.296375 | 2.872844 | 0.001201 |
| AL355574.1 | 1.81282 | 1.343125 | 2.446769 | 0.000101 |
| AP001065.1 | 0.675793 | 0.551892 | 0.827509 | 0.000149 |
