# Supplementary material for: Identification of a Ferroptosis-Related Long Noncoding RNA Prognostic Signature and Its Predictive Ability to Immunotherapy in Hepatocellular Carcinoma
Source: Front Genet. 2021 Oct 21;12:682082. doi: 10.3389/fgene.2021.682082 (PMC8566703; doi:10.3389/fgene.2021.682082)
Supplement: Supplementary file 4 [file Table3.docx]

| Ferroptosis Gene | lncRNA | correlation | P value | Regulation |
| --- | --- | --- | --- | --- |
| AKR1C2 | LUCAT1 | 0.505283722 | 1.25E-25 | positive |
| FTL | LUCAT1 | 0.406934447 | 2.38E-16 | positive |
| AKR1C3 | LUCAT1 | 0.545992265 | 1.92E-30 | positive |
| G6PD | LUCAT1 | 0.400535949 | 7.63E-16 | positive |
| TXNRD1 | LUCAT1 | 0.657661307 | 1.08E-47 | positive |
| MAFG | LUCAT1 | 0.472968799 | 3.04E-22 | positive |
| NQO1 | LUCAT1 | 0.445541556 | 1.23E-19 | positive |
| PRDX1 | LUCAT1 | 0.454918664 | 1.67E-20 | positive |
| SQSTM1 | LUCAT1 | 0.525516983 | 6.10E-28 | positive |
| AKR1C1 | LUCAT1 | 0.666709518 | 2.00E-49 | positive |
| SRXN1 | LUCAT1 | 0.62258686 | 1.61E-41 | positive |
| PGD | LUCAT1 | 0.573949691 | 3.76E-34 | positive |
| SLC1A5 | AC099850.3 | 0.524828821 | 7.36E-28 | positive |
| BRD4 | AC099850.3 | 0.459538387 | 6.14E-21 | positive |
| BACH1 | AC099850.3 | 0.405148656 | 3.31E-16 | positive |
| RRM2 | AC099850.3 | 0.719511776 | 7.17E-61 | positive |
| SLC2A1 | AC099850.3 | 0.414625396 | 5.69E-17 | positive |
| EIF2S1 | AC099850.3 | 0.477013542 | 1.20E-22 | positive |
| ATG16L1 | AC099850.3 | 0.40032996 | 7.92E-16 | positive |
| HMGB1 | AC099850.3 | 0.508160012 | 5.99E-26 | positive |
| VDAC2 | AC099850.3 | 0.418234538 | 2.87E-17 | positive |
| DNAJB6 | AC099850.3 | 0.417556951 | 3.27E-17 | positive |
| PIK3CA | AC099850.3 | 0.543903429 | 3.52E-30 | positive |
| MAPK9 | AC099850.3 | 0.40542787 | 3.14E-16 | positive |
| SP1 | AC099850.3 | 0.540850152 | 8.46E-30 | positive |
| STMN1 | AC099850.3 | 0.644019441 | 3.39E-45 | positive |
| ATG7 | AC099850.3 | 0.425595281 | 6.92E-18 | positive |
| MAPK3 | AC099850.3 | 0.433370118 | 1.48E-18 | positive |
| G6PD | AC099850.3 | 0.443384756 | 1.92E-19 | positive |
| ELAVL1 | AC099850.3 | 0.544165342 | 3.26E-30 | positive |
| MAPK8 | AC099850.3 | 0.472823222 | 3.14E-22 | positive |
| CDKN2A | AC099850.3 | 0.450379951 | 4.42E-20 | positive |
| HIF1A | AC099850.3 | 0.495557776 | 1.43E-24 | positive |
| MAFG | AC099850.3 | 0.440838434 | 3.25E-19 | positive |
| YY1AP1 | AC099850.3 | 0.447823878 | 7.59E-20 | positive |
| NRAS | AC099850.3 | 0.614292104 | 3.59E-40 | positive |
| OXSR1 | AC099850.3 | 0.540918647 | 8.30E-30 | positive |
| AURKA | AC099850.3 | 0.51375746 | 1.40E-26 | positive |
| IREB2 | AC099850.3 | 0.422123625 | 1.36E-17 | positive |
| ATG3 | AC099850.3 | 0.530131964 | 1.72E-28 | positive |
| TFRC | AC099850.3 | 0.462263431 | 3.37E-21 | positive |
| PML | AC099850.3 | 0.446322996 | 1.04E-19 | positive |
| FANCD2 | AC099850.3 | 0.73168658 | 6.82E-64 | positive |
| BECN1 | AC099850.3 | 0.420918512 | 1.72E-17 | positive |
| PRKAA1 | AC099850.3 | 0.426644339 | 5.63E-18 | positive |
| SETD1B | AC099850.3 | 0.434818117 | 1.11E-18 | positive |
| HELLS | AC099850.3 | 0.638852331 | 2.77E-44 | positive |
| MAPK1 | AC099850.3 | 0.515113991 | 9.84E-27 | positive |
| ABCC1 | AC099850.3 | 0.435469076 | 9.71E-19 | positive |
| NF2 | AC099850.3 | 0.419734838 | 2.15E-17 | positive |
| ATG5 | AC099850.3 | 0.501029708 | 3.66E-25 | positive |
| HIF1A | AL365203.2 | 0.418657923 | 2.65E-17 | positive |
| SLC38A1 | AL365203.2 | 0.598292834 | 1.11E-37 | positive |
| ABCC1 | AL365203.2 | 0.460080771 | 5.45E-21 | positive |
| DNAJB6 | AL031985.3 | 0.471018477 | 4.75E-22 | positive |
| NCF2 | AL031985.3 | 0.405967209 | 2.85E-16 | positive |
| STMN1 | AL031985.3 | 0.446488239 | 1.01E-19 | positive |
| MAPK8 | AL031985.3 | 0.402472477 | 5.38E-16 | positive |
| NRAS | AL031985.3 | 0.538278511 | 1.76E-29 | positive |
| ATG3 | AL031985.3 | 0.459050124 | 6.83E-21 | positive |
| FANCD2 | AL031985.3 | 0.444384211 | 1.56E-19 | positive |
| MAPK1 | AL031985.3 | 0.416719622 | 3.83E-17 | positive |
| ATF4 | AC009005.1 | 0.417610195 | 3.23E-17 | positive |
| DDIT3 | AC009005.1 | 0.404207387 | 3.93E-16 | positive |
| OTUB1 | AC009005.1 | 0.40138593 | 6.55E-16 | positive |
